# Photoinactivation of *Mycobacterium tuberculosis* and *Mycobacterium smegmatis* by Near-Infrared Radiation Using a Trehalose-Conjugated Heptamethine Cyanine

**DOI:** 10.3390/ijms25158505

**Published:** 2024-08-04

**Authors:** Nataliya V. Kozobkova, Michael P. Samtsov, Anatol P. Lugovski, Nikita V. Bel’ko, Dmitri S. Tarasov, Arseny S. Kaprelyants, Alexander P. Savitsky, Margarita O. Shleeva

**Affiliations:** 1 A.N. Bach Institute of Biochemistry, Federal Research Centre ‘Fundamentals of Biotechnology’ of the Russian Academy of Sciences, Moscow 119071, Russia; 2 A.N. Sevchenko Institute of Applied Physical Problems of the Belarusian State University, 220045 Minsk, Belarus

**Keywords:** *Mycobacterium tuberculosis*, mycobacteria, antibacterial photodynamic inactivation, trehalose, dormant mycobacteria, tricarbocyanine dye

## Abstract

The spread of multidrug-resistant mycobacterium strains requires the development of new approaches to combat diseases caused by these pathogens. For that, photodynamic inactivation (PDI) is a promising approach. In this study, a tricarbocyanine (TCC) is used for the first time as a near-infrared (740 nm) activatable PDI photosensitizer to kill mycobacteria with deep light penetration. For better targeting, a novel tricarbocyanine dye functionalized with two trehalose units (TCC2Tre) is developed. The photodynamic effect of the conjugates against mycobacteria, including *Mycobacterium tuberculosis*, is evaluated. Under irradiation, TCC2Tre causes more effective killing of mycobacteria compared to the photosensitizer without trehalose conjugation, with 99.99% dead vegetative cells of *M. tuberculosis* and *M. smegmatis*. In addition, effective photoinactivation of dormant forms of *M. smegmatis* is observed after incubation with TCC2Tre. Mycobacteria treated with TCC2Tre are more sensitive to 740 nm light than the Gram-positive *Micrococcus luteus* and the Gram-negative *Escherichia coli*. For the first time, this study demonstrates the proof of principle of in vitro PDI of mycobacteria including the fast-growing *M. smegmatis* and the slow-growing *M. tuberculosis* using near-infrared activatable photosensitizers conjugated with trehalose. These findings are useful for the development of new efficient alternatives to antibiotic therapy.

## 1. Introduction

Most pathogenic mycobacteria cause lung infections, although in some cases lymph nodes, bones and joints, skin and wounds are affected [1]. However, the most concerning disease caused by mycobacteria is tuberculosis (TB), caused by *Mycobacterium tuberculosis*. The situation with this disease is complicated by the high and increasing levels of antibiotic-resistant strains of *M. tuberculosis* and multidrug-resistant tuberculosis resistant to first-line antibiotics (isoniazid and rifampicin). Chemotherapy treatment for such infections has extremely limited effectiveness [2].

A special aspect of this problem is the ability of pathogenic mycobacteria to form dormant forms to survive under stress conditions, including in the host body. In this state, bacteria stop all biosynthetic processes, including those that are targeted by antibiotics, and thus become invulnerable to the action of antimicrobial drugs. *M. tuberculosis* in a dormant state is the cause of latent tuberculosis—a widespread, asymptomatic, and difficult-to-treat form [3]. Such forms of mycobacteria are able to remain viable for a long time in the human body before entering an active state, causing the resumption of the disease, for example, in the treatment of coronavirus inflammation using corticosteroids [4]. 

The traditional direction for combating drug resistance is associated with the search for new antibiotic substances. Modern methods of identifying new targets in pathogen cells, deciphering their structures and selecting active substances using screening systems have significantly increased the efficiency of such studies. However, within a very short period of time from the start of using a new antibiotic in the clinic, resistant strains arise. Such cycles of developing new drugs and the subsequent emergence of resistant strains can be repeated indefinitely.

The combination of these challenges necessitates the development of fundamentally novel approaches to combat genetically determined resistant strains and dormant forms of mycobacteria.

Antimicrobial photodynamic therapy (aPDT), or photodynamic inactivation (PDI), represents an interesting alternative or adjunct to chemotherapy for microbiological control [5]. This method reduces systemic toxicity, is non-invasive, minimizes antibiotic use and mitigates the risk of resistance due to its broad action on biomolecules. aPDT is a promising method for addressing various bacterial infections, particularly those that are antibiotic-resistant [6]. Currently, methylene blue, radachlorin and various porphyrin derivatives are used as exogenous photosensitizers (PSs) for the inactivation of mycobacteria [5]. Mycobacterial cells with endogenous accumulated porphyrines also exhibit a great sensitivity to illumination [7,8,9]. Such PSs are activated by light with a wavelength not exceeding 690 nm, which prevents their widespread use for inactivating the pathogen in the deep tissues of the macroorganism. While the application of aPDT is not as widespread for mycobacteria as for other bacterial species, studies have shown that it can effectively deactivate various mycobacterial strains, especially those that cause skin infections, in both laboratory settings and animal models [5]. Despite the fact that the inclusion of exogenous PS leads to a high inactivation effect in vitro, the use of chemically synthesized PSs is complicated by their transportation to localized foci of bacterial infections. This obviously limits the use of PDI to superficial (skin) diseases.

Consequently, a promising avenue of research is the application of new PSs activated in the near-infrared range to kill mycobacteria, which should increase the depth of the effective action of light. The rationale for this assumption is the use of tricarbocyanine (TCC) dyes activated in the near-infrared range (780 nm), which has been demonstrated to increase the depth of tumor damage [10]. However, until now, such PSs have not been used for the PDI of mycobacteria.

The second challenge when using exogenous PSs to inactivate mycobacteria located inside the host body is the potential for side effects on host tissues. When conducting aPDT using hydroxyaluminum phthalocyanine (Photosens) as a PS in an experimental model of tuberculosis infection in mice, it was demonstrated that the tissues of the experimental animal retained a significant amount of light energy. Nevertheless, the residual light transmission was sufficient to provide a pronounced therapeutic effect, manifested in a significant reduction of the mycobacterial load and specific inflammatory process in all examined internal organs [11]. However, side effects on the host organism were observed when using light with a high dose density (above 100 J/cm^2^). While it effectively inactivates *M. tuberculosis*, this illumination regimen is unacceptable for clinical purposes, as it causes severe photonecrosis of photosensitized tissues [12]. 

To enhance the specificity of PSs targeting bacteria, the conjugation of fluorescent molecules with antibiotics, peptides and antibodies was employed [13]. In particular, trehalose-related PSs for the PDI of mycobacteria were synthesized and studied [14,15].

Trehalose is an essential component for mycobacteria [16] and serves as a precursor for various glycolipids in their cell envelopes [17]. Mycobacteria are capable of producing trehalose internally but can also use trehalose from external sources. Indeed, mycobacteria have an effective trehalose transporter, which is localized in the cell wall. This transporter can transport trehalose and trehalose analogues, including conjugates with PSs, from the external environment into the bacterium [15,18]. Because trehalose is not produced by mammals, the basis for selectivity of trehalose-linked PS uptake by bacterial cells within a macroorganism is established [18]. It was shown that ^14^C-trehalose can effectively enter and be absorbed by mouse macrophages and is subsequently taken up by *M. tuberculosis* bacteria within those cells [19]. This result illustrates the potential of trehalose-functionalized probes for in vivo applications.

In this study, we report a synthetic strategy to prepare a conjugate of a tricarbocyanine (heptamethine cyanine) dye with trehalose and evaluate its photodynamic effect against mycobacteria (slow-growing *M. tuberculosis* and fast-growing *M. smegmatis*) in different physiological states to demonstrate proof of principle.

## 2. Results

**Figure 1:**
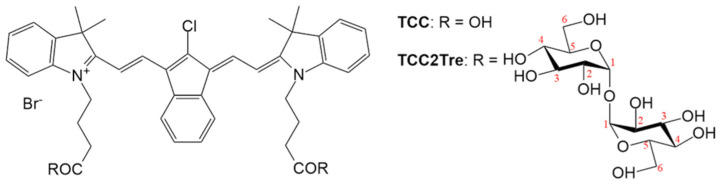
The structural formulae of TCC and TCC2Tre. The numbers of carbon atoms in trehalose are indicated in red. Each OH group of trehalose could form a link with TCC. The molar masses for TCC and TCC2Tre (bromide salts) are 742 and 1389 g/mol, respectively.

The conjugates with trehalose were obtained using a previously synthesized tricarbocyanine dye (TCC) in the form of a bromide salt bearing 4-meso-chloride and an indene moiety in the polymethine chain (Figure 1) [10]. To prepare the conjugates of TCC with trehalose (TCC2Tre), both carboxyl groups of TCC were esterified with trehalose (Figure 1) using 1-methyl-2-bromo-1-methylpyridiniummethanesulfonate as an analogue of the Mukaiyama reagent [20]. The synthesized TCC2Tre, according to HPLC and MS analysis, was a mixture of six isomers due to the ability of trehalose to form esters at different carbon atoms. The predominant isomer was the diester bonded at the sixth carbon atom in both trehalose units (for more details and analytical data, see Section 4.2 and Figures S1–S4). In the following experiments, this mixture of isomers is referred to as “TCC2Tre”.

### 2.1. The Binding of TCC2Tre and TCC with Mycobacterium smegmatis Cells

The interaction of TCC2Tre and TCC with *M. smegmatis* was investigated by analyzing the spectral properties of the dyes bound with mycobacterial cells. *M. smegmatis* is a non-pathogenic relative of the causative agent of tuberculosis. The species was chosen for these experiments as it is a generally accepted model for the study of mycobacteria including *M. tuberculosis* [21].

**Figure 2:**
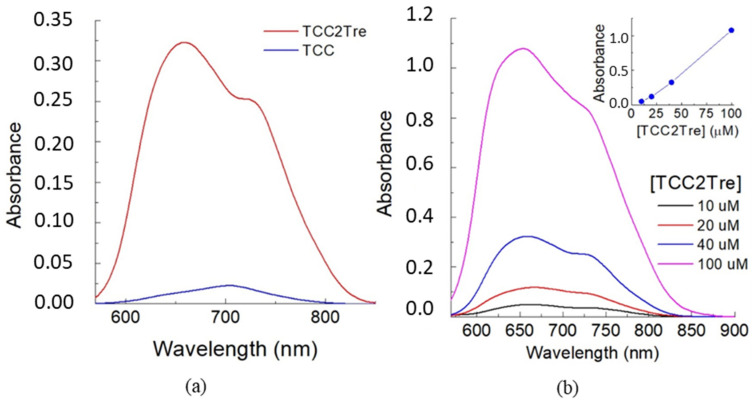
Absorption spectra for the suspensions of *M. smegmatis* cells in PBS incubated with TCC2Tre and TCC. (**a**) Equal amounts of bacterial cells (with the final concentration of 10^7^ cells/mL corresponding to the absorbance of 0.2 at 590 nm) were incubated with 40 µM TCC2Tre (red curve) and TCC (blue). After washing, the cells were resuspended in PBS to the concentration of 10^7^ per ml, and the spectra were recorded. (**b**) The cells were incubated with 10 µM (black curve), 20 µM (red curve), 40 µM (blue curve) and 100 µM (magenta curve) TCC2Tre for 1 h in PBS. After washing, the cells were resuspended in PBS to the concentration of 10^7^ per ml, and the spectra were recorded. The inset shows peak absorbance as a function of dye concentration.

First, TCC2Tre and TCC were compared with respect to their binding with *M. smegmatis*. The mycobacterial cells were incubated with the dyes in PBS for 1 h and then washed to remove unbound dye molecules. As illustrated in Figure 2a, the absorbance of TCC2Tre bound with mycobacteria exceeds that of TCC by an order of magnitude. As anticipated, the incorporation of trehalose into the molecular structure of TCC markedly enhances its binding with mycobacterial cells. The absorbance of TCC2Tre bound with *M. smegmatis* is almost directly proportional to the concentration of the dye between 10 and 100 µM in the incubation medium (Figure 2b).

**Figure 3:**
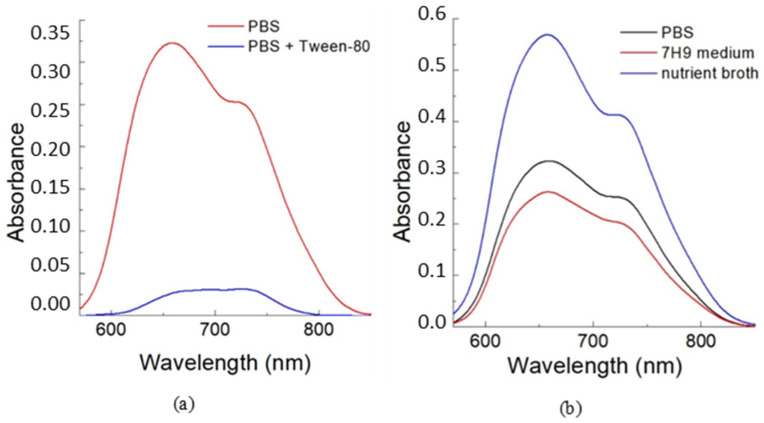
Absorption spectra for the suspensions of *M. smegmatis* in PBS incubated with TCC2Tre in different media. The cells were incubated with 40 µM TCC2Tre for 1 h. After washing, the cells were resuspended in PBS to the concentration of 10^7^ per ml, and the spectra were recorded. (**a**) The cells were incubated in PBS (red curve) and in PBS with 0.05 wt% Tween-80 added (blue curve). (**b**) The cells were incubated in PBS (black curve), 7H9 mineral medium (red curve) and the peptide-rich NB medium (blue curve).

Tween-80 is frequently added to growth media for mycobacteria to prevent their aggregation [22]. The impact of this detergent on the binding of TCC2Tre with *M. smegmatis* was also examined. As shown in Figure 3a, the presence of 0.05 wt% Tween-80 during the incubation period results in a significant decrease in the absorbance of TCC2Tre bound to *M. smegmatis*, by approximately one order of magnitude. The detergent probably competes for the dye in PBS, thus reducing the affinity of the dye for the cell wall.

In addition to PBS, *M. smegmatis* were also incubated with TCC2Tre in 7H9 mineral (broth base) medium and in rich nutrient broth (NB). After the incubation period, the mycobacteria were washed from unbound dye and resuspended in PBS. While the binding of TCC2Tre with *M. smegmatis* is similar in PBS and 7H9 medium, it is improved approximately 2-fold in the NB medium (Figure 3b).

### 2.2. The Comparison of the Photoinactivation of M. smegmatis Cells with TCC and TCC2Tre

The effectiveness of TCC and TCC2Tre (Figure 1) for the photoinactivation of *M. smegmatis* cells using 740 nm light was compared. It was found that the use of the hydrophobic TCC results in almost zero effectiveness of *M. smegmatis* photoinactivation (Table 1). However, the incubation of the cells with TCC in the presence of BSA improved photodynamic activity. Presumably, the adsorption on BSA prevented the aggregation of the hydrophobic TCC thus allowing the dye to maintain its properties. A similar effect was produced by the addition of Tween-80 (0.05%) during the incubation of the cells with TCC (Table 1).

**Figure 4:**
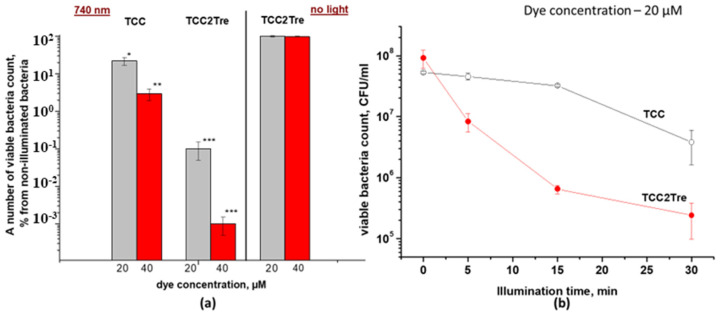
The survival of *M. smegmatis* cells after light exposure. *M. smegmatis* cells were cultured and subjected to PDI as described in Section 4. The incubation of bacteria with the dyes was performed in the NB medium in the presence of 5% BSA. Following exposure to 740 nm light at an irradiance of 240 mW/cm^2^ under static conditions, 100 μL of the cell suspension was plated on agar plates for the CFU estimation. (**a**) A 2 h incubation period with various concentrations of the dyes was followed by illumination for 30 min; 100% corresponds to the viability of non-illuminated bacteria incubated without dyes. (**b**) The cells were incubated with 20 µM of the dyes followed by illumination under static conditions for various time intervals. Each experiment was repeated three times. The bars represent ± standard deviation. Asterisks indicate that the results are significantly different from the control (no light) by Student’s *t*-test. *p*-values are indicated as follows: * *p* < 0.05. ** = *p* < 0.01, *** = *p* < 0.001.

Therefore, the photodynamic effects of the two dyes were compared under identical conditions, where BSA was added to NB medium for binding. The results of these experiments are presented in Figure 4.

TCC demonstrated a rather weak photodynamic effect against *M. smegmatis*. At a starting concentration of 40 μM TCC and a light dose of 468 J/cm^2^, the number of dead *M. smegmatis* cells was 97%. At the same concentration and light dose, the photoactivity of TCC2Tre was substantially higher, with the number of dead *M. smegmatis* cells reaching 99.999% (Figure 4a). These data on the photoinactivation of *M. smegmatis* are consistent with the data on the binding of these PSs with mycobacteria (Figure 2). At a dye concentration of 40 μM, the dark toxicity of these compounds against *M. smegmatis* was completely absent (Figure 4a, Table 1). Furthermore, the illumination of vegetative cells of *M. smegmatis* at a maximum irradiance of 240 mW/cm^2^ for 30 min without prior incubation with the dyes did not result in a significant decrease in bacterial viability (Table 1).

At an irradiance of 240 mW/cm^2^, the maximum inactivation of *M. smegmatis* cells with TCC2Tre was achieved after 15 min of illumination. Further increase in the exposure time did not significantly improve the photoinactivation effect (Figure 4b). With TCC and at the same irradiance, noticeable mycobacterial death was observed after 30 min of illumination (Figure 4b).

**Figure 5:**
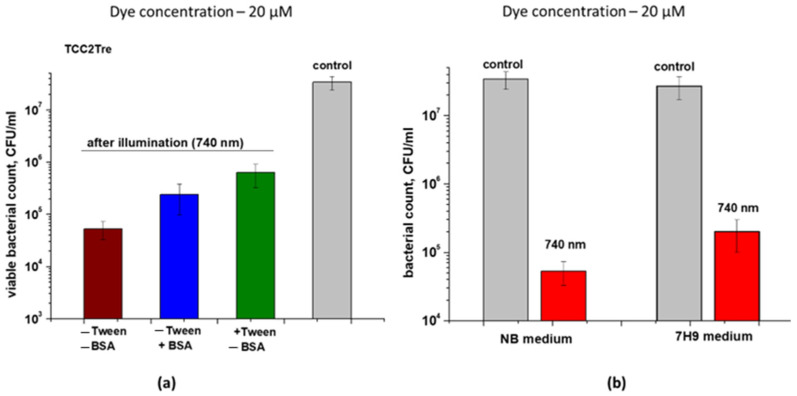
The influence of medium composition on TCC2Tre effectivity. *M. smegmatis* cells were cultured and subjected to PDI as described in Section 4. Following exposure to 740 nm light at an irradiance of 240 mW/cm^2^ under static conditions, 100 μL of the cell suspensions was plated on agar plates for CFU estimation. (**a**) Incubation with 20 µM TCC2Tre in the presence or absence of 5% BSA and 0.05% Tween-80 for 2 h at 37 °C in the NB medium. (**b**) A 2 h incubation with 20 µM TCC2Tre in different media without Tween-80 and BSA was followed by illumination for 30 min. Each experiment was repeated two times. The bars represent ± standard deviation.

In contrast to TCC, the presence of BSA or Tween-80 significantly reduced the photoactivity of TCC2Tre against *M. smegmatis* cells (Figure 5a). This is consistent with the negative influence of Tween-80 on the binding of TCC2Tre with the cells (Figure 3a). Thus, the maximum photodynamic effect was observed when TCC2Tre was used in the absence of both BSA and Tween-80 in the medium (Figure 5a). Nevertheless, even in the presence of BSA, the photoinactivation of *M. smegmatis* using TCC2Tre was more effective than using TCC (Figure 4).

To assess the influence of environmental components on the photoactivity of TCC2Tre for *M. smegmatis* cells, two media were tested: rich NB and mineral medium 7H9. The incubation of the bacteria with TCC2Tre in the NB medium resulted in an improved photodynamic effect compared to the mineral medium (Figure 5b).

Thus, the optimal conditions for achieving the maximum photodynamic effect of the water-soluble TCC2Tre against *M. smegmatis* cells are as follows: incubation in a rich medium without Tween-80 or BSA. In contrast, for the hydrophobic TCC, the maximum photodynamic effect requires the presence of BSA or Tween-80 in the incubation medium.

**Figure 6:**
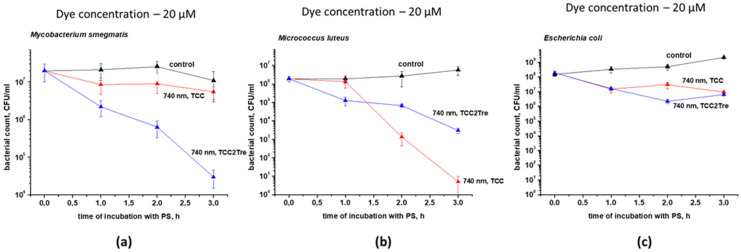
The photoinactivation of bacteria with different cell wall compositions after binding with TCC or TCC2Tre. Bacterial cells were cultured and subjected to PDI as described in Section 4. The incubation with 20 µM of the dyes was performed in the NB medium. The cells were incubated with TCC in the presence of BSA and with TCC2Tre in the absence of BSA. Before illumination, the bacteria were washed two times in PBS. Following exposure to 740 nm light at an irradiance of 240 mW/cm^2^ under static conditions, 100 μL of the cell suspensions were plated on agar plates for CFU estimation. (**a**) *Mycobacterium smegmatis*; (**b**) *Micrococcus luteus* (Gram-positive); (**c**) *Escherichia coli* (Gram-negative). The control samples were non-illuminated cells incubated with the PSs. Each experiment was repeated three times. The bars represent ± standard deviation.

The influence of the incubation time of *M. smegmatis* cells with the PSs on the photoinactivation was studied (Figure 6a). The photodynamic effect was found to be proportional to the incubation time with TCC2Tre in the NB medium. More specifically, a 3 h incubation period resulted in a decrease in the CFU number by almost three orders of magnitude. TCC exhibited low activity even when incubated with BSA under the same conditions and for the same duration.

The effect of the new TCC2Tre against bacteria with different cell wall structures was assessed. *Micrococcus luteus* was used as a representative of Gram-positive bacteria, *Escherichia coli* was employed as a representative of Gram-negative bacteria and *M. smegmatis* was selected as a representative of mycobacteria. It was found that TCC2Tre is more active against *M. smegmatis*, and TCC is more active against *M. luteus* (Figure 6a,b). Both dyes were almost ineffective against *E. coli* (Figure 6c).

**Figure 7:**
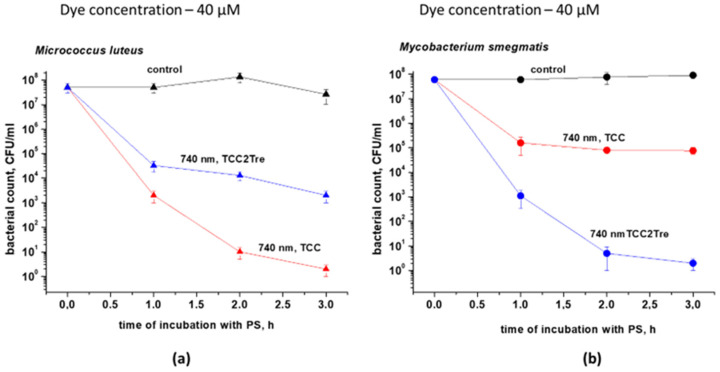
The photoactivity of TCC and TCC2Tre in a 1:1 mixed culture containing *Micrococcus luteus* and *Mycobacterium smegmatis*. Bacterial cells were cultured and subjected to PDI as described in Section 4. The incubation with 40 µM of the dyes was performed in the NB medium without Tween-80 for 1, 2 and 3 h. The incubation medium for TCC contained BSA, while the incubation medium for TCC2Tre did not contain BSA. Following exposure to 740 nm light at an irradiance of 240 mW/cm^2^ under static conditions, 100 μL of the cell suspensions were plated on agar plates for CFU estimation. (**a**) *Micrococcus luteus*; (**b**) *Mycobacterium smegmatis*. The control samples were non-illuminated cells incubated with the PSs. Each experiment was repeated two times. The bars represent ± standard deviation.

The effectiveness of these PSs was assessed in a mixed 1:1 culture consisting of *M. smegmatis* and *M. luteus*. The distinct morphology and coloration of the colonies in conjunction with the low bacterial concentration of both species allowed us to evaluate the effect of the PSs on each bacterial species simultaneously. TCC was found to be more effective against *M. luteus*, while TCC2Tre was more active against mycobacteria, even in the context of a mixed culture comprising the two microbial species (Figure 7).

**Figure 8:**
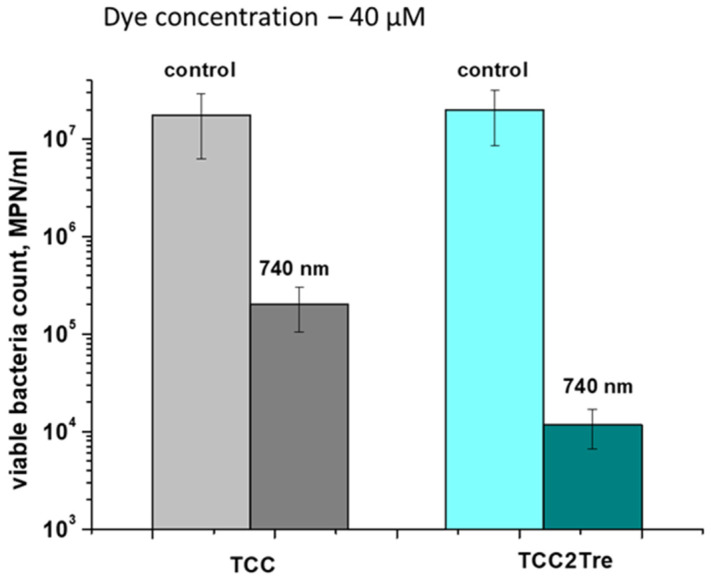
PDI of cultures of *M. smegmatis* cells in a dormant state (6 months). Dormant *M. smegmatis* cells were obtained and subjected to PDI as described in Section 4. The bacteria were incubated with 40 µM of the dyes in Sauton medium in the presence of Tween-80 for 2 h. The incubation medium for TCC contained BSA, while the incubation medium for TCC2Tre did not contain BSA. Following exposure to 740 nm light at an irradiance of 240 mW/cm^2^ for 30 min under static conditions, the viability of the bacteria was estimated by the MPN assay in liquid medium. The MPN method was performed for two biological replicates, with three series of dilutions made within each. The bars represent ± standard deviation.

The effectiveness of TCC2Tre against the dormant form of *M. smegmatis* was also assessed. The dormant form obtained under conditions of gradual acidification of the external environment developed “non-culturability” (absence of growth on solid media) which requires MPN assay for the estimation of their viability [23]. As in the case of vegetative cells of *M. smegmatis*, incubation of dormant cells (6 months of dormancy) of *M. smegmatis* with TCC2Tre or TCC allowed photodynamic inactivation of these forms of bacteria although at a lesser extent than for viable vegetative cells (Figure 8).

**Figure 9:**
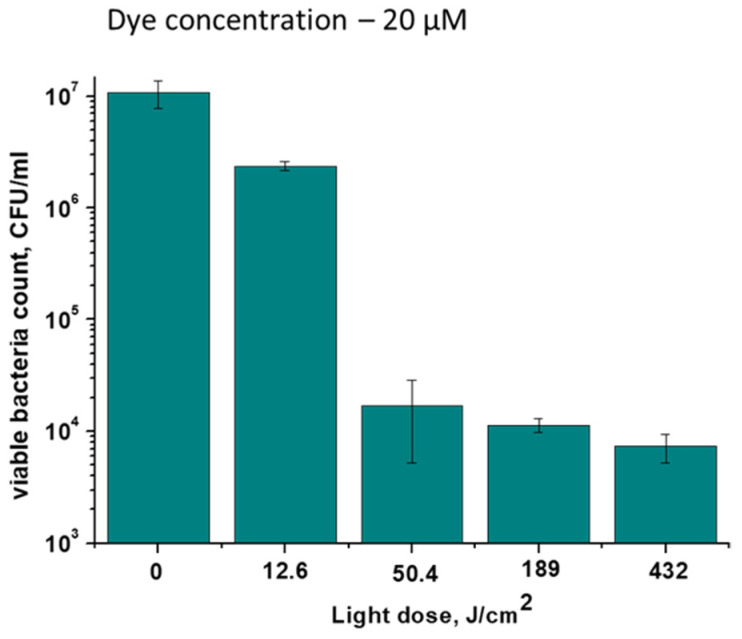
The effect of light dose on the efficiency of photoinactivation of *M. smegmatis* in the presence of TCC2Tre. Bacterial cells were cultured and subjected to PDI as described in Section 4. The bacteria were incubated with 20 µM TCC2Tre in the NB medium without Tween-80 for 2 h at 37 °C, washed and exposed to 740 nm light at an irradiance of 240 mW/cm^2^ for varying periods of time. Each experiment was repeated three times. A typical experiment is shown. The bars represent ± standard deviation.

The minimum light dose required for effective photoinactivation of *M. smegmatis* using TCC2Tre was determined. At the same irradiance (240 mW/cm^2^) and different exposure times, the optimal light dose for the inactivation of *M. smegmatis* was between 50 and 180 J/cm^2^ (Figure 9). A further increase in the light dose did not greatly affect the number of viable mycobacteria. At a light dose of 50 J/cm^2^, significant inactivation of viable bacteria was observed, while a light dose of approximately 12 J/cm^2^ was ineffective (Figure 9). For comparison, a light dose of 100 J/cm^2^ was previously demonstrated to be well tolerated by mouse tissues during photodynamic therapy with an aluminum phthalocyanine PS [12]. 

### 2.3. Efficiency of the TCC2Tre for the Photoinactivation of Mycobacterium tuberculosis

**Figure 10:**
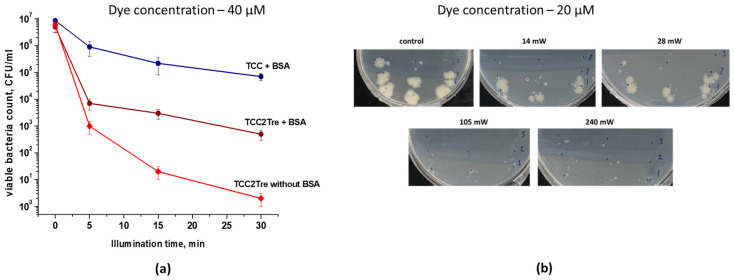
Photoinactivation of *M. tuberculosis* cells after their incubation with TCC or TCC2Tre. Bacterial cells were cultured and subjected to PDI as described in Section 4. The incubation with the dyes was performed in the 7H9 medium without Tween-80 for 2 h at 37 °C. TCC was prepared with BSA, while TCC2Tre was prepared with and without BSA. Before illumination, the bacteria were washed two times in PBS. (**a**) The viability of *M. tuberculosis* cells incubated for 2 h with 40 µM of the dyes and exposed to 740 nm light at an irradiance of 240 mW/cm^2^ for varying periods of time. The experiment was repeated three times. The bars represent ± standard deviation. (**b**) The effect of the irradiance value on the efficiency of photoinactivation of *M. tuberculosis* in the presence of TCC2Tre. Cells were incubated in the 7H9 medium for 2 h with 20 µM TCC2Tre without Tween-80 and BSA. Then, the bacteria were washed and exposed to 740 nm light at a light dose of 100 J/cm^2^ and different irradiances (mW/cm^2^) as described in Section 4.6. The plates were incubated at 37 °C for 22 days. The experiment was repeated two times.

The sensitivity of the *M. tuberculosis* H37Rv strain to PDI in the presence of tricarbocyanine PSs was assessed. Similar to the experiments with *M. smegmatis* (Figure 4), TCC2Tre was more effective against *M. tuberculosis* compared to TCC (Figure 10a). In the absence of BSA, the activity of TCC2Tre increased (Figure 10a). The PDI of *M. tuberculosis* in the presence of TCC2Tre is more pronounced compared to the PDI of *M. smegmatis* under the same conditions (Figure 4 and Figure 10a). With increasing illumination time at an irradiance of 240 mW/cm^2^, an increase in the number of dead cells in the *M. tuberculosis* culture was observed. At a light dose of 468 J/cm^2^, complete inactivation of *M. tuberculosis* cells was revealed when using TCC2Tre incubated without BSA (Figure 10a).

The effect of irradiance on the photoinactivation of *M. tuberculosis* cells was assessed at a constant light dose of 100 J/cm^2^. The most effective inactivation of *M. tuberculosis* was observed at irradiances above 100 mW/cm^2^ (Figure 10b). 

## 3. Discussion

By selectively targeting specific molecules, light-active compounds have the ability to address limitations in drug discovery. Progress in synthesizing and testing photosensitizer conjugates with biomolecules like antibodies, peptides or small-molecule drugs is resulting in more effective agents for eliminating a wide range of microbial species [24]. 

In the present study, we sought to combine the spectral advantages of TCC for deeper light penetration of tissues upon aPDT with trehalose conjugation (Figure 1) for improved targeting of mycobacteria. To this end, to demonstrate proof of principle we synthesized novel trehalose conjugates of TCC (TCC2Tre) in the form of a mixture of six isomers, which are activated by light at a wavelength of 740 nm. The obtained isomers were formed because trehalose units bonded to TCC at a different carbon atoms with a predominant isomer of the sixth carbon atom in two trehalose units. We attempted to use OH-protected trehalose, more specifically, its hexa(trimethylsilyl) derivative with free hydroxyl groups at the sixth position. However, the trimethylsilyl groups were removed by cesium fluoride. The synthesis of pure TCC2Tre isomers requires the development of other synthetic strategies, which is currently underway. At same time, the trehalose mycolyltransesterase enzyme Ag85A, which transports trehalose into mycobacterial cells, revealed very broad substrate specificity processing equally well chemically modified trehalose (but not monosaccharides) [19]. In interactome study, the lipoprotein LpqN was identified as a direct or indirect interacting partner of the mycolyltransferases FbpA and FbpB, as well as of the periplasmic domains of MmpL3 and MmpL11. LpqN also interacts with secreted cell envelope biosynthetic enzymes such as Ag85A via pulldown assays. The X-ray crystal structures of LpqN and LpqN bound to dodecyl-trehalose suggest that LpqN directly binds trehalose ester, the MmpL3 and Ag85A substrate. The crystal structures of LpqN in complex with trehalose-6-decanoate and dodecyl trehalose suggest that LpqN may directly bind different trehalose esters and thus serve as a facilitator of their periplasmic translocation [25]. As a result, we might expect that all synthesized isomers are taken up by mycobacterial cells. Naturally, the synthesis and/or separation of individual active isomers and their study is necessary for their further application in pharmacology.

As expected, TCC2Tre demonstrated superior binding with *M. smegmatis* cells in comparison with unconjugated TCC (Figure 2). Correspondingly, the PDI of *M. smegmatis* with TCC2Tre was more pronounced than that with TCC (Figure 4 and Figure 6a). The specificity of TCC2Tre for mycobacteria was evident from the comparison of its photoactivity against bacteria with different cell wall structures (Figure 6). This was confirmed in the experiment with a mixed culture, where TCC2Tre was substantially more effective against *M. smegmatis* than against *M. luteus* (Figure 7). 

In two previous publications, the photoactivity of different PSs conjugated with trehalose towards mycobacteria was demonstrated. To improve the performance of photodynamic therapy for *M. smegmatis* and *M. abscessus*, PSs linked to trehalose (2AT2AT-PPIX; 6AT-I-BODIPY) were specifically designed. These compounds are able to penetrate the mycomembrane more effectively, resulting in a stronger photodynamic effect when exposed to light at a wavelength of 550–650 nm. These compounds exhibited minimal dark toxicity and were well tolerated by mammalian cells [14]. In another study, a trehalose-porphyrin conjugate with a two-carbon linker (C2) was shown to display increased phototoxicity against *M. smegmatis* following exposure to 415 nm light in comparison to the conjugate with a six-carbon linker (C6) [26].

Trehalose conjugates of some fluorescent compounds were also used for the detection of *M. tuberculosis*. Synthetic analogs of trehalose and trehalose monomycolate have demonstrated the ability to be incorporated into mycomembrane components in a specific and metabolically relevant manner. This enables their analysis in their natural environment and provides new opportunities for the targeted detection and treatment of mycobacterial pathogens within complex biological systems [27,28,29,30]. In particular, the fluorogenic dye DMN-Tre, a conjugate of 4-N,N-Dimethylamino-1,8-naphthalimide and trehalose, selectively labels the mycobacterial cell wall, making it a valuable tool for studying *M. tuberculosis* physiology both in vitro and intracellularly [31,32]. A novel trehalose probe based on a 3-hydroxychromone (3HC-3) dye was developed. When attached to trehalose, this dye exhibits a 10-fold increase in fluorescence intensity compared to DMN-Tre [33]. A fluorescent probe cephalosphorinase-dependent green trehalose (CDG-Tre) was constructed to specifically label single live Bacille Calmette-Guérin (BCG) cells within macrophages. CDG-Tre demonstrated high selectivity for mycobacteria compared to other species in the *Corynebacterineae* suborder [30]. Selective targeting of mycobacteria was also achieved using trehalose derivatized carbazole (Tre-Cz). The presence of trehalose in the conjugate with carbazole allowed for the detection of mycobacteria in mixed cultures and in patient sputum [34]. Given that trehalose conjugates have the ability to reach the periplasmic space in mycobacteria, it is possible to design small molecules that can specifically bind to transmembrane protein domains for targeted immobilization. This strategy is not restricted to mycobacteria but can potentially be applied to various pathogens [35].

In one of the aforementioned experiments, a trehalose conjugate with BODIPY was found to have a minimum inhibitory concentration of 0.5–33 µM for its photoactivity against *M. smegmatis* and *M. abscessus* according to the resazurin method for estimation of viability [14]. However, for the trehalose-porphyrin conjugate, only one concentration of the compound was used (10 mM), which resulted in a discernible inhibition of *M. smegmatis* growth according to the resazurin method [26]. In our experiments, we determined the effect of PDI by counting cell viability via CFU, which directly estimates the bactericidal effect in contrast to the resazurin method. We found that 20 μM TCC2Tre was sufficient for photoinactivating 99.9% of mycobacteria (Figure 4a). The discrepancy in the effective concentrations in the current work and previous publications may be attributed to different efficiency of PSs and the methodologies employed for phototoxicity and cell viability assessment.

TCC2Tre at a concentration of 40 µM produced significant killing effect on *M. tuberculosis* cells (Figure 10a). To the best of our knowledge, this represents the first instance of the application of a trehalose-conjugated PS for the PDI of the pathogenic *M. tuberculosis*.

In the present study, the incubation periods of mycobacteria with the PSs were relatively short (2–3 h). It is evident that in mineral media such as PBS or Middlebrook base, in the absence of essential nutrients (e.g., glycerol, glucose, etc.), only a limited amount of TCC2Tre is capable of binding to the cell wall without being consumed by the cells during this period (Figure 3b). At the same time, a significant increase in the quantity of bound TCC2Tre in the rich NB medium in comparison with the mineral media may be attributed to the uptake of the PS by cells within the first hour of incubation. Similarly, an increase in cell sensitivity to light during incubation with TCC2Tre but not TCC in the NB medium (Figure 6a and Figure 7b) may also reflect active uptake of TCC2Tre by cells. Active accumulation of trehalose-bound substances by mycobacterial cells and their incorporation in mycolic acids via a specific trehalose transporter over a 24 h incubation period was experimentally proven [14,31,32,33,34].

The significantly lesser sensitivity of dormant *M. smegmatis* cells for the PSs and the lesser difference in sensitivity between TCC2Tre and TCC could be due to the sufficiently depressed metabolism in dormant cells [23] and suppression of transport processes. Nevertheless, the dormant cells still exhibited photosensitivity (Figure 8), which could be due to the hydrophobic interaction of the dye molecules with the cell wall structure.

In conclusion, our work demonstrates the possibility of using PSs targeted to the cell wall of mycobacteria and activated in the near-infrared range, which expands the horizons for the aPDT of intracellular pathogens such as *Mycobacterium tuberculosis*. The application and study of the new PSs for in vivo PDT using laboratory animals are currently underway in our laboratory.

## 4. Materials and Methods

### 4.1. Bacterial Strains, Growth Media, and Culture Conditions

Strains *Mycobacterium smegmatis* mc2 155, *Escherichia coli* K-12 and *Micrococcus luteus* NCIMB 13267 (previously described as Fleming strain 2665) were grown aerobically in 20 mL of the peptide-rich nutrient broth (NB) medium (Himedia, Mumbai, India) for 20 h at 37 °C under constant shaking at 110 rpm. *M. smegmatis* were grown with Tween-80 (0.05%) added to the medium.

Strain *Mycobacterium tuberculosis* H37Rv was grown in Middlebrook 7H9 liquid medium (Himedia, India) with supplements (ADC and 0.05% Tween-80) for 7 days at 37 °C, under constant shaking at 180 rpm. 

### 4.2. Synthesis of a Tricarbocyanine Dye Functionalized with Trehalose (TCC2Tre) and Its Characterization

TCC is a bromide salt of a hydrophobic 4-meso-chloro-substituted indotricarbocyanine dye with an indene moiety in the polymethine chain (Figure 1). TCC was synthesized as described in Ref. [10]. To obtain TCC2Tre, both carboxyl groups of TCC were esterified with trehalose (Figure 1) using 1-methyl-2-bromo-1-methylpyridiniummethanesulfonate as an analogue of the Mukaiyama reagent [20]. Anhydrous cesium fluoride (5 equiv.) and 1-methyl-2-bromo-1-methylpyridinium (2 equiv.) were added to a dimethylformamide solution of TCC. A dimethylformamide solution of anhydrous trehalose (4 equiv.) was then added to this mixture. After allowing the reaction mixture to stand at room temperature for 10 h, it was diluted with ethyl acetate, and the resulting precipitate was filtered through filter paper and washed with deionized water to remove cesium salts. After filtration, the precipitate was washed with acetone to remove TCC and pyridine compounds and then with ethanol.

The resulting compounds were characterized by high-performance liquid chromatography (HPLC) coupled to electrospray ionization mass spectrometry (ESI-MS). HPLC was performed on an Agilent 1200 LC system equipped with a Poroshell 120 EC-C18 column (4.6 × 50 mm, 2.7 µm). Mobile phase A was 0.05 vol% trifluoroacetic acid and mobile phase B was MeCN. The linear gradient was 40–95% phase B in 0–10 min. Chromatograms were recorded at 710 nm with a photodiode detector. After the chromatographic separation, absorption spectra were recorded using a diode-array detector. ESI-MS analysis was then performed on an Agilent 6410 Triple Quadrupole LC/MS system in positive ion detection mode with the fragmentor voltage set at 135 V.

The chromatogram for the synthesized compounds shows six peaks with retention times of 8.07, 8.19, 8.32, 8.45, 8.70 and 8.86 min (Figure S1). The mass spectra for all these peaks contain species with *m*/*z* = 1309.5 corresponding to the esters of TCC with two trehalose units, i.e., TCC2Tre (Figure S2). The additional peaks in the mass spectra with lower *m*/*z* values can be assigned to the fragments of the molecular ion of TCC2Tre that appear during electrospray ionization. The six species with *m*/*z* = 1309.5 correspond to esters formed between the carboxyl groups of TCC and various hydroxyl groups of trehalose. The primary hydroxyl group located at the sixth carbon atom (see Figure 1) of trehalose is known to have the greatest relative reactivity [36]. Therefore, the most intense HPLC peak with a retention time of 8.19 min should correspond to a diester bonded at the sixth carbon atom in both trehalose units. The HPLC peaks with retention times of 8.07 and 8.32 min are more intense compared to the rest of the peaks and can therefore be assigned to TCC esters with one of the trehalose units bonded at the sixth carbon atom and the other trehalose unit bonded at a different carbon atom. The absorption spectra corresponding to the six HPLC peaks are almost identical in position and shape (Figure S3).

The chemical structure of TCC2Tre was also confirmed using ^1^H nuclear magnetic resonance (NMR) spectroscopy (Figure S4). NMR spectra were as acquired in MeOD using an AVANCE 500 (Bruker) spectrometer at 25 °C and 500 MHz. The ^1^H NMR spectra for TCC2Tre, TCC and trehalose are shown in Figure S4.

TCC2Tre: ^1^H NMR (500 MHz, MeOD) δ 8.45—8.01 (m, 4H), 7.67–6.98 (m, 12H), 5.56–4.99 (m, 8H), 4.45–3.40 (m, 16H), 2.75 (t, *J* = 36.7 Hz, 4H), 2.33–1.90 (m, 4H), 1.85–1.56 (m, 12H).

### 4.3. Binding of Tricarbocyanine Dyes with Mycobacterial Cells

An overnight culture of *M. smegmatis* was centrifuged (5 min at 8000 rpm), and the supernatant was discarded. The pellet was resuspended in PBS (50 mM, pH 7.45, Gibco, New York, NY, USA), the NB medium, or Middlebrook 7H9 mineral medium. The absorbance of the resulting cell suspensions at 590 nm was 0.2, which corresponded to ~10^7^ cells/mL. *M. smegmatis* were incubated at 37 °C with different concentrations (5, 10, 20, 40 and 100 µM) of TCC2Tre and TCC. The dyes were introduced into cell suspensions via a DMSO solution, with the final concentration of DMSO in cell suspensions being 5 vol%. Following a 1, 2 or 3 h incubation period, the mycobacterial cells were washed with fresh PBS and centrifuged at 8000 rpm for 5 min three times. Following this, the cells were resuspended in PBS to a concentration of 10^7^ cells/mL and transferred to 1 cm semi-micro polystyrene cuvettes (Fisher Scientific, Pittsburgh, PA, USA, catalog No. 11547692). Absorption spectra of the cell suspensions were acquired using a SOLAR PV1251 spectrophotometer.

### 4.4. Preparation of the Dormant Forms of M. smegmatis upon the Medium Self-Acidification

*Mycobacterium smegmatis* mc^2^155 cells were initially grown in the NB medium at 37 °C for 24 h, with shaking at 200 rpm. The inoculum (1 mL) was added to 100 mL of Sauton medium, containing (g/L) K_2_HPO_4_, 0.5; MgSO_4_·7H_2_O, 1.4; L-Asparagine, 4.0; ferric ammonium citrate, 0.05; Na_3_(citrate), 2.0; ZnSO_4_, 0.001 (Sigma Aldrich, Burlington, VT, USA), and 0.2 vol% glycerol (Panreac, Barcelona, Spain). The initial pH value was 6.0 (instead of pH 7.0 typical of the usual Sauton composition). To prevent aggregation of the cells, Tween-80 was added (0.05%). The culture was grown at 37 °C under constant stirring (200 rpm) for 18 days until a constant pH value (ca. 6.0) of the medium was established [23]. Dormant cells were stored for 6 months without agitation in 15 mL capped tubes in the dark.

### 4.5. Preparation of Bacteria for Photoinactivation

The bacterial cell cultures were diluted with sterile culture medium to the desired absorbance. The absorbance was measured in 1.5 mL plastic cuvettes with an optical path length of 10 mm using a photoelectric colorimeter (Biochrom WPA CO7500 Colourwave, Biochrom, Cambridge, UK). The culture medium was used as a control. One mL of cell suspension (0.1 absorbance at 590 nm) and 10 µL of a 4 mM TCC (or TCCTre) solution were added to a sterile 2 mL tube. Bacteria were incubated with the dyes for different time intervals at 37 °C under constant stirring. Following the incubation period, the cells were centrifuged for 10 min at 10,000 rpm. The precipitate was washed two times with PBS for 10 min at 10,000 rpm.

### 4.6. Antibacterial Photoinactivation 

Suspensions of bacteria with 0.1 absorbance at 590 nm, which corresponds to ca. 10^7^ bacteria per mL, were used for light inactivation experiments. Aliquots (100 µL) of the bacterial suspensions were placed in the wells of a 96-well plate (Nunc). Illumination was carried out using a 740 nm LED SOLIS-740C (Thorlabs, Newton, NJ, USA) with a bandwidth of 45 nm (full width at half maximum). The light beam was collimated to a diameter of 5 mm, corresponding to the diameter of the wells of a 96-well plate. Illumination coupling MT-CON with optimized optics for bright bottom homogenous illumination of a suspension in the wells was used. The irradiance in each mode of the LED was measured with a Newport 2936-c power meter (Table 2). In most cases, a light dose of 468 J/cm^2^ (240 mW/cm^2^ irradiance and 30 min illumination time) was used. For some experiments, a light dose of 100 J/cm^2^ was used, which was obtained with a different irradiance (Table 2). The temperature in was controlled with a Fluke 80BK Type K Multimeter Thermocouple Temperature Probe (Fluke Corporation, Everett, Washington, DC, USA) directly in the microwells before and after illumination and in the presence and without dyed *M. smegmatis* suspension with ±0.2 °C precision. The temperature was lower than 40 °C in the wells during all the experiments.

After illuminating the samples, serial tenfold dilutions (10^−1^ to 10^−7^) were prepared in the NB medium with 0.05% Tween-80, and aliquots (3 × 20 μL) were plated on NB agar plates for CFU (for vegetative bacteria) or MPN (for dormant bacteria) determination.

### 4.7. Viability Estimation by CFU

For *M. smegmatis*, *E. coli*, and *M. luteus*, bacterial suspensions were serially diluted in fresh NB medium, and then three replicate 10 μL samples from each dilution were spotted on NB (Himedia, India) agar. The plates were incubated at 37 °C for 3–5 days, then the number of colony forming units (CFUs) was counted. 

For *M. tuberculosis*, bacterial suspensions were serially diluted in fresh 7H9 medium (Himedia, India) containing 0.05% Tween-80, and then three replicate 10 μL samples from each dilution were spotted on 7H10 (Himedia, India) agar. Plates were incubated at 37 °C for 21 days, and then the number of colony forming units (CFUs) was counted. The limit of detection was 10 CFU/mL.

### 4.8. MPN Assay

The MPN procedure was performed for dormant *M. smegmatis* cells in 48-well plastic plates (Corning Inc., Corning, NY, USA) filled with a 1:1 mixture of Sauton medium and the NB medium. Appropriate serial dilutions of *M. smegmatis* cells (100 μL) were added to each well. The plates were incubated at 37 °C with agitation (130 rpm) for 14 days. Wells with the visible bacterial growth were counted as positive, and the MPN values were calculated using standard statistical methods [37,38]. These experiments were performed two times in three technical replicas.

### 4.9. Statistics

Statistical processing was carried out via the analysis of the standard deviation or relative error within each data group. All the data are presented as the mean values of three replicate determinations. The MPN values were determined using de Man’s tables calculated on the basis of a Poisson distribution [30]. For the MPN assay, (95%) confidence limits were calculated [38]. The MPN values were considered statistically different if low and high confidence limits were not overlapping. Student’s test assuming unequal variance was performed for estimation of significance for comparative data. *p*-values are indicated as follows: * = *p* < 0.05, ** = *p* < 0.01, *** = *p* < 0.001.

## Figures and Tables

**Table 1:** The influence of the composition of the incubation medium on the photodynamic activity of TCC2Tre and TCC against *M. smegmatis.*

Incubation Conditions	TCC	TCC2Tre
NB medium	(2.6 ± 1.5) × 10^7^ *	(4 ± 2) × 10^4^ *
NB medium + 5% BSA	(3 ± 2) × 10^5^ *	(1.6 ± 1.5) × 10^5^ *
NB medium + 0.05% Tween-80	(5 ± 3) × 10^5^ *	(5 ± 2) × 10^5^ *
Control with dye without illumination	(3 ± 1.8) × 10^7^ *	(4.6 ± 2) × 10^7^ *

A 2 h incubation period with 20 µM TCC2Tre or TCC was followed by a 30 min illumination at 740 nm at an irradiance of 240 mW/cm^2^. * CFU (cells per mL) for cells after illumination are shown. Control without dye with illumination—(2.3 ± 2) × 10^7^ cells per mL. Control without dye and without illumination—(2 ± 1.2) × 10^7^ cells per mL.

**Table 2:** Irradiance and illumination time used to obtain a constant light dose.

Irradiance (mW/cm^2^)	Illumination Time (min)	Light Dose (J/cm^2^)
240	7	100
105	16	100
28	60	100
14	120	100

## Data Availability

Data are contained within the article or Supplementary Materials.

## Supplementary Materials

The following supporting information can be downloaded at: https://www.mdpi.com/article/10.3390/ijms25158505/s1.
