# Supplementary material for: Photoinactivation of Mycobacterium tuberculosis and Mycobacterium smegmatis by Near-Infrared Radiation Using a Trehalose-Conjugated Heptamethine Cyanine
Source: Int J Mol Sci. 2024 Aug 4;25(15):8505. doi: 10.3390/ijms25158505 (PMC11313374; doi:10.3390/ijms25158505)
Supplement: Supplementary file 1 [file ijms-25-08505-s001.zip › ijms-3084835-supplementary.pdf]

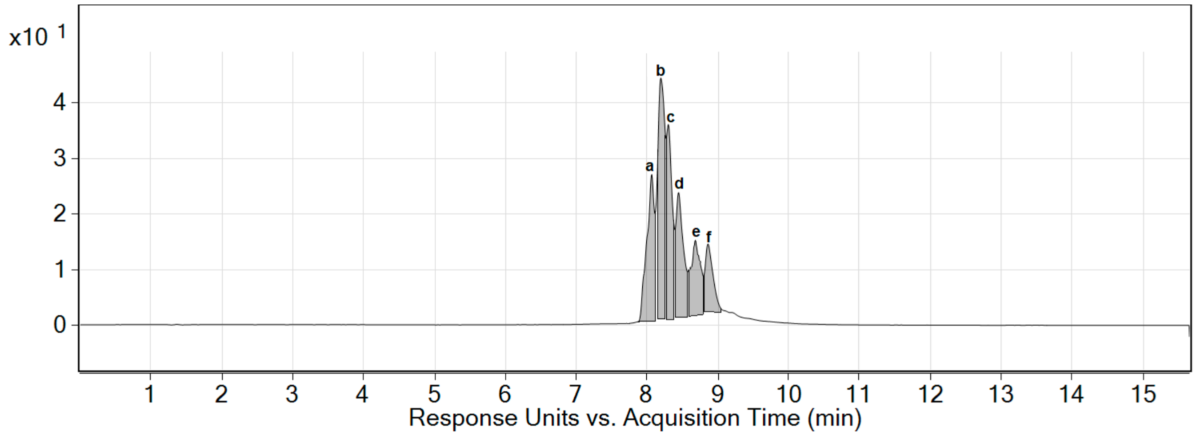

Figure S1. Chromatogram for the synthesized TCC2Tre.

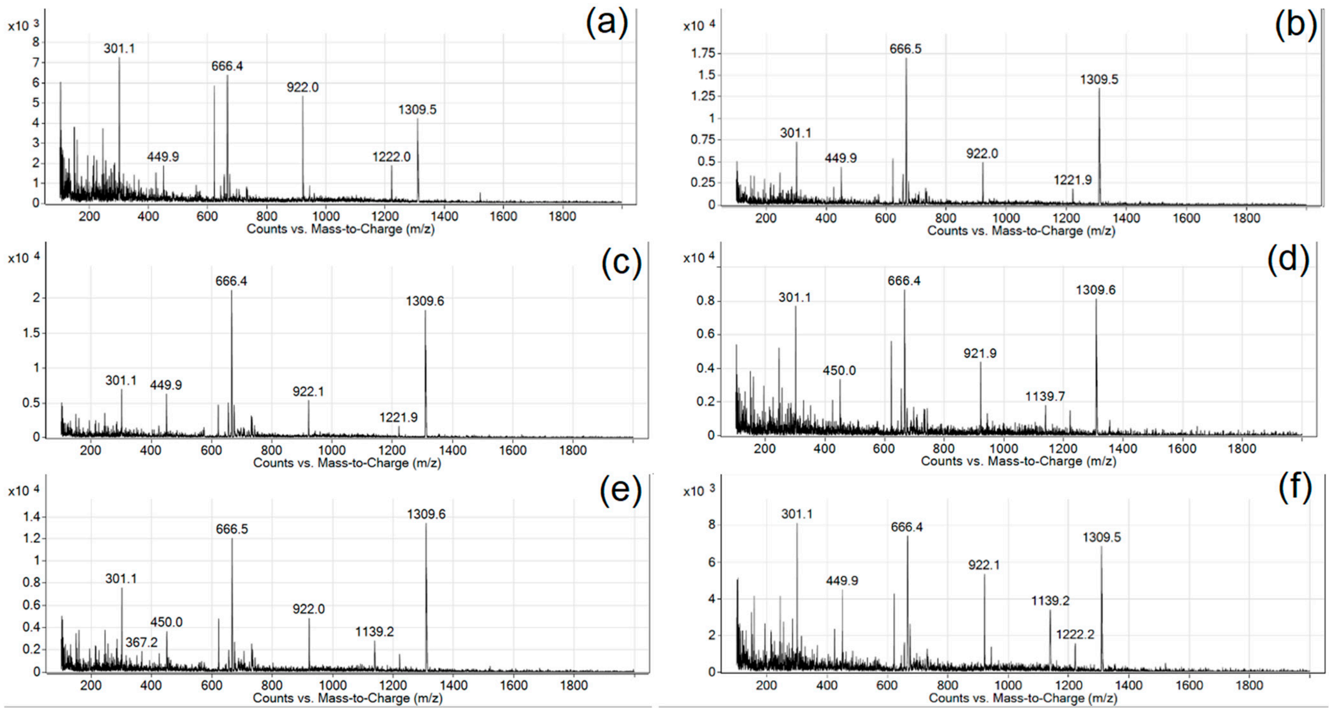

Figure S2. Mass spectra for HPLC peaks **a**–**f** shown in Figure S1. m/z = 1309.5 corresponds to the molecular cation of TCC2Tre.

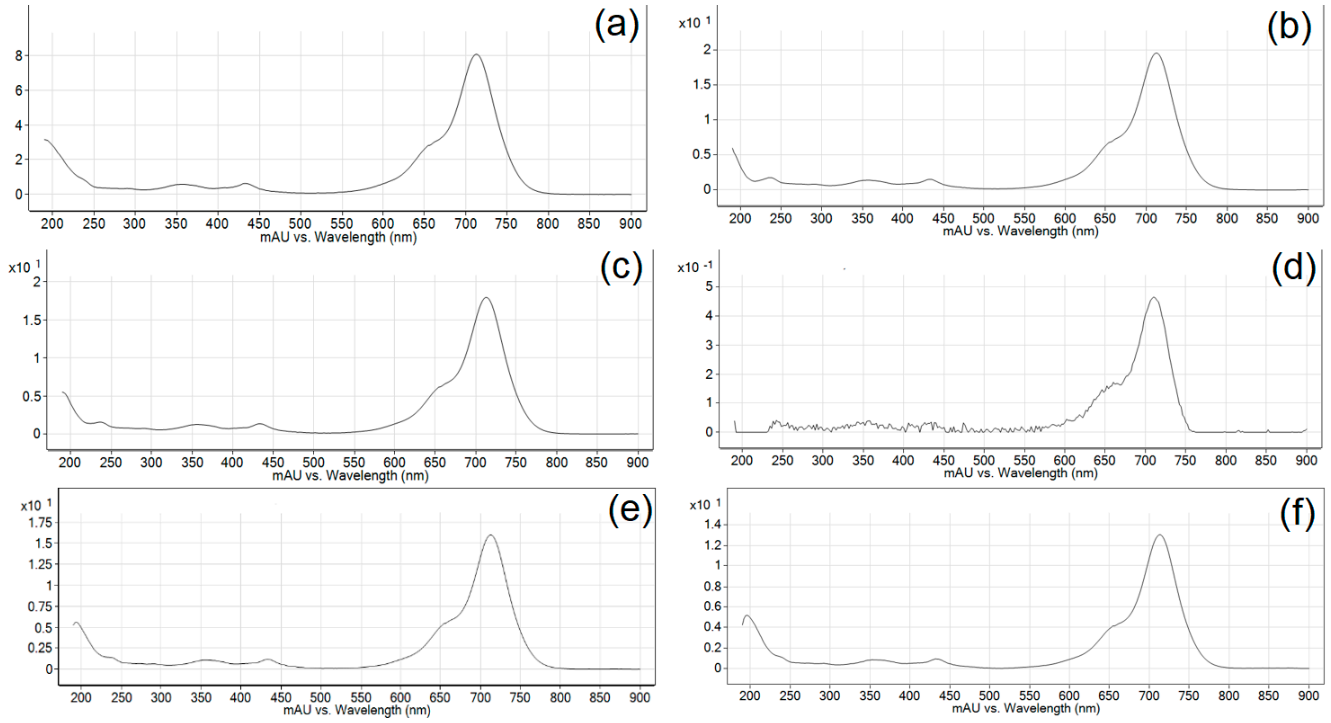

Figure S3. Absorption spectra for HPLC peaks **a**–**f** shown in Figure S1.

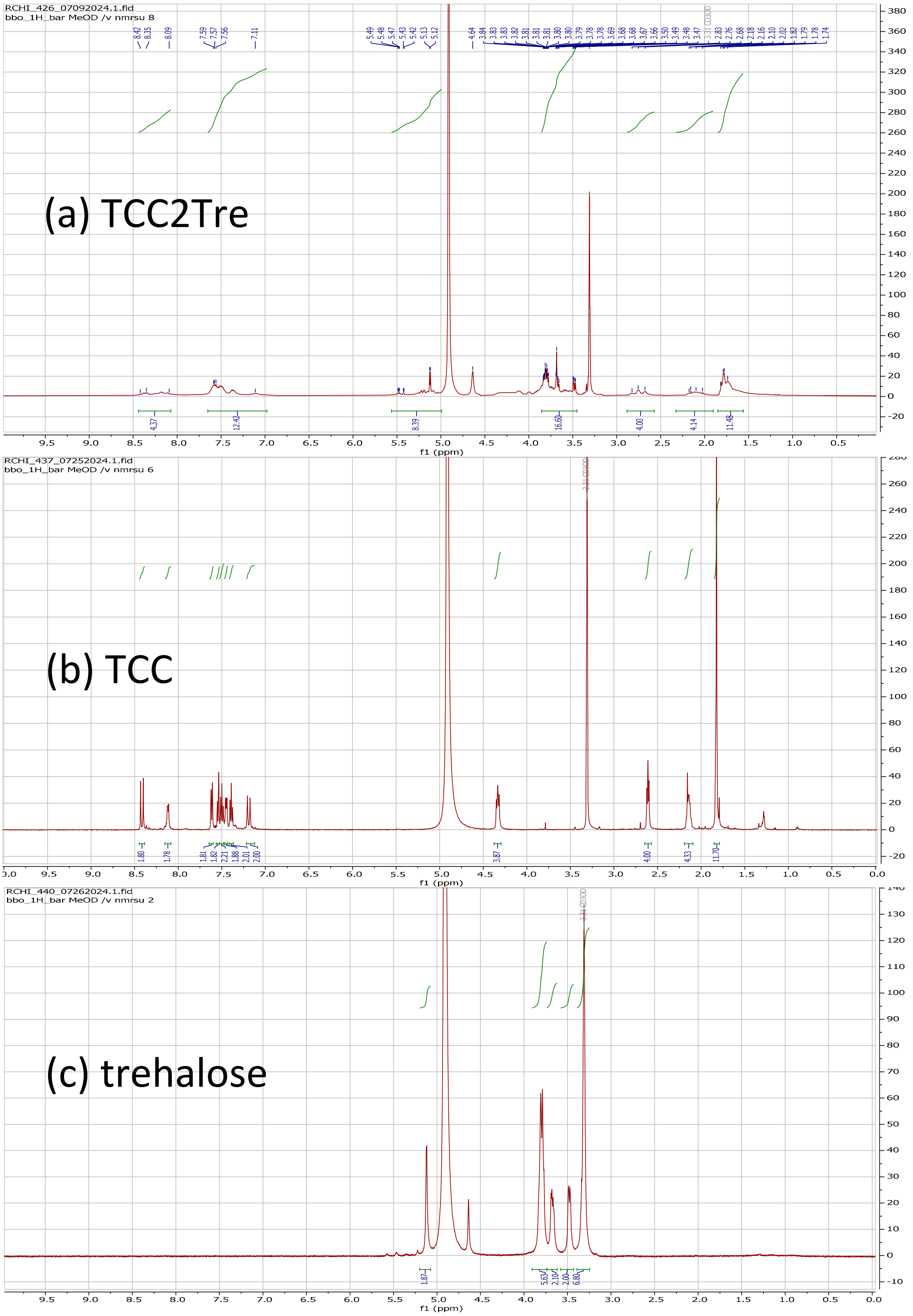

Figure S4. ^{1}H NMR spectra for the TCC2Tre (a), TCC (b), and trehalose (c) acquired in MeOD.
